# Viral taxonomy derived from evolutionary genome relationships

**DOI:** 10.1371/journal.pone.0220440

**Published:** 2019-08-14

**Authors:** Tyler J. Dougan, Stephen R. Quake

**Affiliations:** 1 Department of Physics, Stanford University, Stanford, California, United States of America; 2 Departments of Bioengineering and Applied Physics, Stanford University and Chan Zuckerberg Biohub, Stanford, California, United States of America; Keele University Faculty of Natural Sciences, UNITED KINGDOM

## Abstract

We describe a new genome alignment-based model for understanding the diversity of viruses based on evolutionary genetic relationships. This approach uses information theory and a physical model to determine the information shared by the genes in two genomes. Pairwise comparisons of genes from the viruses are created from alignments using NCBI BLAST, and their match scores are combined to produce a metric between genomes, which is in turn used to determine a global classification using the 5,817 viruses on RefSeq. In cases where there is no measurable alignment between any genes, the method falls back to a coarser measure of genome relationship: the mutual information of 4-mer frequency. This results in a principled model which depends only on the genome sequence, which captures many interesting relationships between viral families, and which creates clusters which correlate well with both the Baltimore and ICTV classifications. The incremental computational cost of classifying a novel virus is low and therefore newly discovered viruses can be quickly identified and classified. The model goes beyond alignment-free classifications by producing a full phylogeny similar to those constructed by virologists using qualitative features, while relying only on objective genes. These results bolster the case for mathematical models in microbiology which can characterize organisms using only their genetic material and provide an independent check for phylogenies constructed by humans, considerably faster and more cheaply than less modern approaches.

## Introduction

Collectively, viruses display an unstructured diversity which hinders the imposition of any systematic classification [1]. Because viruses evolve quickly and lack an analogue to the bacterial 16S sequence, there is no consensus surrounding their taxonomy [2]. The Baltimore classification groups viruses into seven categories based on the biochemistry of their replication strategies, nucleotide character, but as the basis for a phylogeny, it conflicts with the observation that some viruses with similar functions and structural proteins have different types of genomes [3]. The International Committee on the Taxonomy of Viruses (ICTV) uses a hierarchical taxonomy inspired by the modern tree of life. However, because this classification has been built up over time in order to accommodate new discoveries, it enjoys neither the tree of life's coherence nor its authority [4,5]. When a new virus is discovered, subjective analysis is required in order to incorporate it into the taxonomy. Other classifications have been proposed based on structure, host species, or genome length [6].

The viral genome captures a record of fingerprints of the evolutionary history of the virus and should in principle provide the basis for calculating relationships between any set of viruses. Pedulla et al. have shown that the genome encodes history including recombination events and virus-species coevolution [7]. The use of full genomes in viral classification, now possible because of modern sequencing techniques, offers the promise of weighing all salient features of a given virus more objectively than the ICTV's consensus system. Indeed, expert consensus has highlighted the need for the ICTV classification to incorporate viruses known only from metagenomic information, which would require categories to be defined in terms of genetics, as opposed to phenomenology [8]. As of this writing, there are 5,817 full viral genomes on RefSeq and in recent years several researchers have proposed alignment-free methods due to the computational complexity of sequence alignment and the rate at which new viruses are sequenced [9,10]. Typically a vector in a Euclidean “genome space'' is calculated from various properties of the genome, and distances between such vectors are used for classification. These include Yu et al. (2013) and Hoang et al. (2015) [11,12]. Although they provide an injective mapping onto the chosen genome space, it is not clear that the distance between two points in genome space is a meaningful metric of genome similarity. More complex models such as GRAViTy have performed very well at classifying new viruses into existing lineages, and even identifying when new viruses do not fit into established families [13]. These models point to the possibility of a completely quantitative top-down classification of viruses—one which, instead of facilitating additions to the ICTV classification, provides an independent check of it.

Alignment-based classifications, such as the one which will be described below, stand on stronger interpretive footing: the “distance” between two genomes can be defined directly from a similarity score returned by an alignment. Gene alignment is most appropriate for sequences that are very similar, and as we will see, a global tree can be built up by considering only these relationships. To find these relationships, we use the sequence alignment tool BLAST; its efficiency not only makes the alignment of 5,817 genomes tractable, but also reduces the laboriousness of re-computing the taxonomy after a significant number of new viruses have been sequenced. Rohwer et al. (2002) propose a phylogeny for phage using BLAST hits; although successful within this more limited scope, their heuristic distance metric makes binary distinctions, reducing the amount of information used [14]. Here, we present the results of a statistically motivated alignment-based phylogeny which considers genomes as collections of individual genes; we find that it correlates well with the ICTV, host kingdom, and Baltimore classifications, and also provides additional insights beyond these.

## Methods

Intuitively, we want to impose a distance metric on the space of viral genomes. From this distance metric, we should be able to extract clustering information, and build a taxonomy. We have sought to find a metric which captures the shared information between genes in a pair of genomes. This approach was inspired by the notion of mutual information from information theory, but uses a more simple measure of shared information based on current flow in parallel resistor networks. In this case information is meant to play the role of current (or, more precisely, information lost in evolution is meant to play the role of resistance) and the more genes that have relationships and the stronger the relationships then the stronger the overall relationship between the genomes is judged to be.

### Gene alignment distance

Functionally, a genome is a collection of genes. This suggests that we begin calculating a distance between genomes by calculating the distances between their component genes. Suppose we have some way to quantify the dissimilarities between the genes in two viruses, and we wish to calculate an overall distance between the viruses as a whole. The overall genome distance between virus A and virus B should satisfy certain properties. If all of the genes in virus A are completely different from all of the genes in virus B, their overall distance should be very large. If virus A and virus B are each composed of a single gene, their overall distance should be equal to the dissimilarity between these two genes. Each additional gene on virus A which is similar to a gene on virus B should only decrease, never increase, the total distance between the viruses.

**Fig 1:**
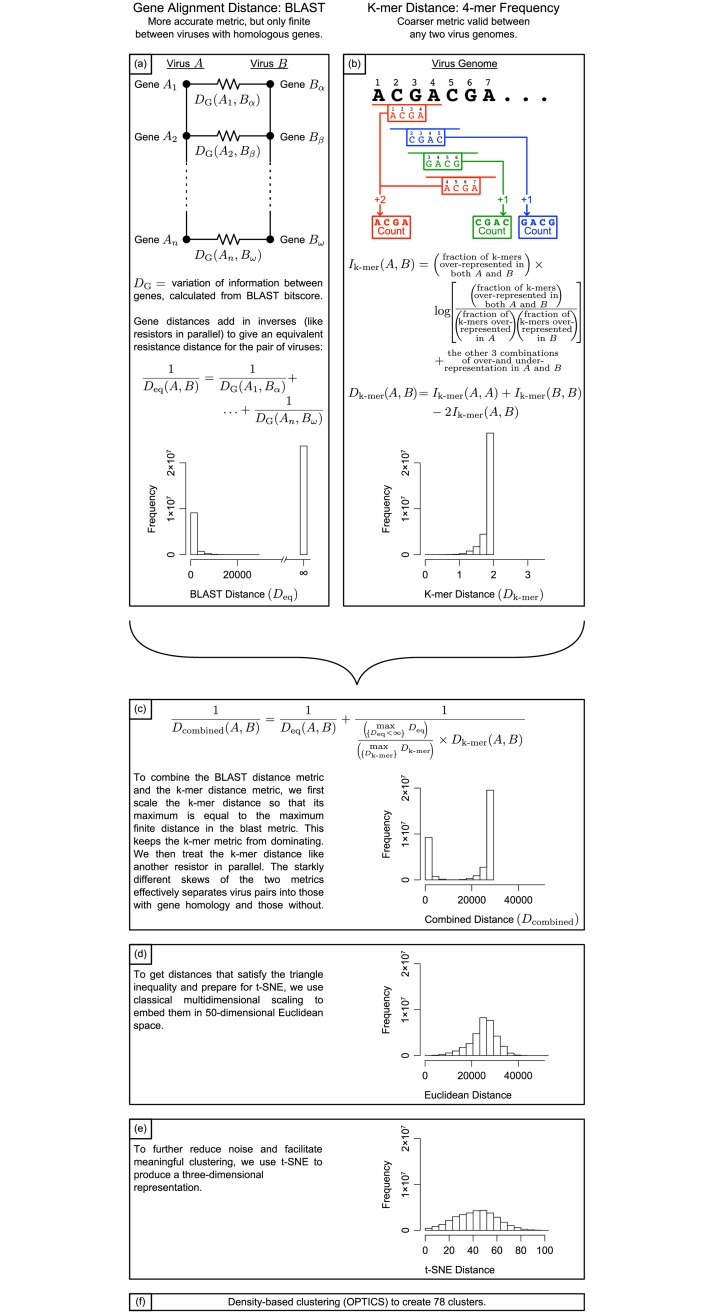
Graphical representation of workflow. (a) Each virus is made up of genes, some of which may match the genes on another virus (Viruses A and B, respectively). We imagine the variation of information between two genes *D*_*i*_ as the resistance of a resistor connecting them. Then the total “distance” between the collections of genes in Viruses A and B is the equivalent resistance between the two sides. Only the genes *A*_*i*_ and *B*_*j*_ which match are shown and indexed; the total number of options for choosing one gene from each virus is quite large, and most such choices do not yield a match. These pairs are analogous to open circuits, with infinite resistance, which do not affect the equivalent resistance. (b) K-mer distance is calculated by assessing whether knowing that a k-mer is over-represented in one genome indicates anything about whether it is over-represented in another genome. (c) The k-mer distance is scaled and then combined with the BLAST distance. (d) Classical multidimensional scaling transforms the distance matrix into a Euclidean position matrix. (e) Three-dimensional embedding with t-SNE allows for clustering and interpretation. (f) Density-based clustering with OPTICS yields 78 clusters, and is the basis for further analysis. Although transformations in (c) and (e) rescale distances, the axes of the histograms are all scaled to correspond with one another.

The above properties are satisfied by the physics of resistors in parallel, suggesting that we calculate the total inverse distance by adding the inverses of the constituent dissimilarities. This analogy is shown in Fig 1(a): if we imagine a resistive wire connecting each of the genes for which a match is found, with resistance equal to the dissimilarity, then the total distance between the genomes is equal to the equivalent resistance between the two. Mathematically, the total distance *D*_eq_ between genomes *A* and *B* with genes *A*_*i*_ and *B*_*j*_ is

$$D_{\text{eq}}=\frac{1}{\sum_{i,j\;\text{genes}\;\text{match}}D_{G}(A_{i},B_{j})}$$ (1)

So given a suitable method for calculating the dissimilarity *D*(*A*_*i*_, *B*_*j*_) between two genes, we can calculate a meaningful distance between two genomes. All that remains is the calculation of dissimilarity between two genes, which requires a measurement of mutual information—a topic which has been well explored as part of the field of information theory [15]. Mutual information, a rigorous measure for the information shared between two variables, is defined as

$$I(A_{i},\;B_{j})=H(A_{i})-H(A_{i}|B_{j})=\sum_{b_{j}\in B_{j}}\sum_{a_{i}\in A_{i}}\text{log}\left(\frac{p(a_{i},b_{j})}{p(a_{i})p(b_{j})}\right)$$ (2)

for two random variables (here, defined by genes) *A*_*i*_ and *B*_*j*_ which take values *a*_*i*_ and *b*_*j*_ (*i* and *j* index the genes, and play no part in the equation; they are retained here only for notational consistency). *I*(*A*_*i*_, *B*_*j*_) is the mutual information between genes *A*_*i*_ and *B*_*j*_, and *H*(*A*_*i*_) and *H*(*A*_*i*_ | *B*_*j*_) are the total and conditional Shannon entropies of genes *A*_*i*_ and *A*_*i*_ given *B*_*j*_, given by $H(A_{i})=\sum_{a_{i}\in A_{i}}p(a_{i})logp(a_{i})$ in analogy with thermodynamic entropy.[15] Then

$$D(A_{i},\;B_{j})=H(A_{i})+H(B_{j})-2I(A_{i},B_{j})$$ (3)

induces a distance metric, known as the variation of information. We will use this metric for our dissimilarity scores between two genes.

Intuitively, the mutual information between two genes should have several features. Two genes which are identical should have a mutual information proportional to their length. Two genes which share nothing in common should have zero mutual information. Each additional bit of mutual information between two genes should represent one additional binary choice which can be correctly determined about one gene, knowing the other. Described another way, each additional bit should represent a doubling of the possible set of random genes from which one gene could be discerned, given knowledge of the other gene.

To calculate the mutual information between genes, we perform sequence alignment using BLAST [8,16–19]. We treat each gene (that is, each coding sequence) as a separate sequence, which results in a database of 300,000 sequences. For each gene, we attempt an alignment of the translated protein sequences against every other sequence using BLAST (TBLASTX), because proteins tend to be more heavily conserved. Protein BLAST translates nucleic acid sequences into amino acids in all six possible reading frames and polarities, which reduces our susceptibility to some database errors. BLAST searches for short match regions between two sequences, and then lengthens these matches to find regions of similarity. Because of a series of simplifications, BLAST is one of the most computationally efficient alignment algorithms available. The BLAST search returns various statistics on the quality of each match; of these, we will concern ourselves with the bit score. For a fixed database, the bit score is a logarithmic function of the e-value, and is linearly related to both the percent match and the alignment length. Moreover, the bit score has a convenient interpretation: it is the size, in bits, of a database one would need to search through to find an equally good match by chance.

It then makes intuitive sense that the bit score should be proportional to mutual information, and indeed this has been shown by Mazandu et al. (2011) [20]. For a fixed database, this proportionality constant varies little between searches; see Korf et al. (2003) for details [21]. Our analysis is unchanged by an overall scaling factor, but this proportionality constant will become important later on. The entropies are determined by the mutual information between a gene and itself. If the mutual information is the amount of information shared between two genes, then the variation of information is the amount of information contained in each virus which is not shared by the other.

Now, given two genes which are found by BLAST to match, we can calculate their dissimilarity using the variation of information; given several such matches between two viruses, we can calculate the distance between the viruses. In the resistor model, gene pairs for which no match is found may be thought of as wires with infinite resistance, or open circuits in parallel.

This formula was used to calculate a distance matrix for all of the viruses considered. Defined distances were found for 30% of the pairs of viruses, and the other 70% of pairs had no matches, because all of their genes were divergent from each other. This was to be expected; alignment fails on highly divergent sequences. Nonetheless, when considering the viruses individually, 70% of the viruses had a match with at least one other virus. Motivated by the need to classify the other 30% of viruses, for which no gene matched any other gene in a virus, we turn to a different genome property from which to calculate a variation of information: k-mer frequency. Ultimately, these two metrics will be combined in order to produce a single comparison between all viruses.

### K-mer distance

It is also possible to calculate the variation of information based on k-mer frequency in genomes, which returns a finite value for all pairs of genomes. K-mer frequency analysis has been a widely applied and useful approach in microbial genome sequencing and in metagenomic analysis [22,23] This is a cruder metric than the BLAST based approach described above, but it enables one to compute mutual information in cases where there are no gene alignments between genomes.

We compare the prevalence of 256 possible k-mers of four letters in each genome (4-mers); 4-mers are short enough to be easily computable, and have been the most successful in genetics tasks such as identifying promoter regions.[24] We consider all reading frames, so the number of 4-mers in a genome is approximately the number of bases, not one-fourth the number of bases. For example, the sequence ACGACGA contains two copies of ACGA, one copy of CGAC, and one copy of GACG (Fig 1(b)). Consider each possible 4-mer as an outcome in the probability theory sense, and build a sigma-algebra out of the power set of 4-mers, so that each event is a unique set of 4-mers between 0 and 256 in size. Define a probability measure on this sigma-algebra which simply gives the fraction of all of the 4-mers in all of the viruses included in the event, so for example, *p*({AAAA, ATAT, ATCG}) = the fraction of 4-mers in all of the viruses combined which are AAAA, ATAT, or ATCG. This gives a probability space. Finally, note that in a given genome, each 4-mer may be more or less prevalent than it is on average in all genetic material in the data set. For a given virus, define a random variable *A* whose input is a given 4-mer and whose output is whether it is over-represented or under-represented in that genome. The random variable *A* can take two possible values *a*: under-representation and over-representation. The probability of over-representation is the fraction of the 256 4-mers which are more prevalent in the given genome than they are in the dataset at large.

This probability space allows for direct calculation of variation of information between two genomes. The mutual information is given by

$$I(A,\;B)=\sum_{b\in B}\sum_{a\in A}p(a,b)\text{log}\left(\frac{p(a,b)}{p(a)p(b)}\right)$$ (4)

This sum has four terms, corresponding to the four combinations of over- and under-representation. If, for example, *a* = *b* = 4-mer over-representation, then *p*(*a*, *b*) is the fraction of 4-mers which are over-expressed in both viruses. If two viruses have correlated distributions of 4-mers, then the joint probabilities of expression in both genomes will be very high; if they have uncorrelated distributions, then representation in one virus will be largely independent of representation in the other, leading to no mutual information. Variation of information can then be calculated as above, providing a finite distance between every two genomes in the data set.

To determine how this distance should be combined with the gene alignment distance above, we note a few general facts. Genes carry the most salient and most conserved information in genomes, so gene alignment, when present, should be considered a better determinant of similarity than 4-mer frequency. K-mer frequency is most relevant when two genomes have no BLAST matches, but for continuity, should contribute to all distances. We would like to treat 4-mer frequency dissimilarity similar to the gene alignment distances—if two viruses are similar in one metric, the distance is low; only if they are dissimilar in both metrics is the distance high. Because it too is a variation of information (a distance metric) measured in bits, the simplest solution is to add the inverse of the 4-mer dissimilarity to the inverse of the gene alignment dissimilarity, effectively treating it as another resistor in parallel.

There remains a problem of magnitudes; the gene alignment distances which are finite range from almost zero to almost 30,000 bits, while the values for the 4-mer variation of information range between zero and two bits. Recall that the BLAST bitscore was only proportional to mutual information, while the 4-mer calculation was exact; we will treat this undetermined proportionality constant as a tunable parameter. In order to prevent the 4-mer distance from eclipsing the gene alignment distance (as a 30 kΩ resistor would become irrelevant in parallel with a 2 Ω resistor), we rescale the 4-mer variation of information so that its maximum is equal to the maximum gene alignment distance. The very strong left skew in the 4-mer distance ensures that when the gene alignment distance is finite, gene alignment dominates in all but five cases out of 10,000,000; in these five, the two distances are comparable (Fig 1(c)).

Although we have argued that the two metrics attempt to measure independent genome features, a sanity check is provided by their consistency. The distances are correlated, albeit nonlinearly. For a given gene alignment distance, there is a lower bound on the 4-mer distance which increases monotonically. Clustering on only one of the two distances reproduces some of the same global features as clustering on the other, but they provide resolution in complementary areas; using only one but not the other is substantially less effective, as shown in S1 and S2 Figs. In particular, the gene alignment distance alone (S1 Fig) clusters well, but the 30% of viruses with no gene matches are unclassifiable: they form a long purple snake of points that winds through the plot (the shape of the snake is specific to t-SNE, which produces the embedding, but the inability to cluster them is not). If only gene sequence alignment is compared, there is no additional information that could be used to find structure within that snake. When we include the k-mer distance, we can break off parts of this snake and find structure in what is left. Conversely, although the k-mer distance alone (S2 Fig) does not leave gaps in information, it does not distinguish well between different magnitudes of similarity (e.g. one thinks intuitively that Zika not just twice as similar to Dengue as it is to torque teno virus, but 100 or 1000 times more similar, yet the k-mer variation of information between Zika and torque teno virus is only twice that between Zika and the Dengue strains). As a result, the data in S2 Fig do not cluster as easily, and the clusters that do form replicate established classifications much less effectively.

### Building a phylogeny

The above methods produce a distance matrix, but further statistical procedures are needed before it can be used for a phylogeny. BLAST's efficiency relies upon several heuristics, and in some cases, these heuristics lead to unphysical situations, such as underestimating the full entropy of a gene. These were dealt with as simply as possible, e.g. by raising negative values to small positive values, in order to avoid dividing by zero. A further issue was that because of BLAST's heuristics and the derived nature of the distance metric used, the distance matrix does not satisfy the triangle inequality, and is overall very noisy.

**Fig 2:**
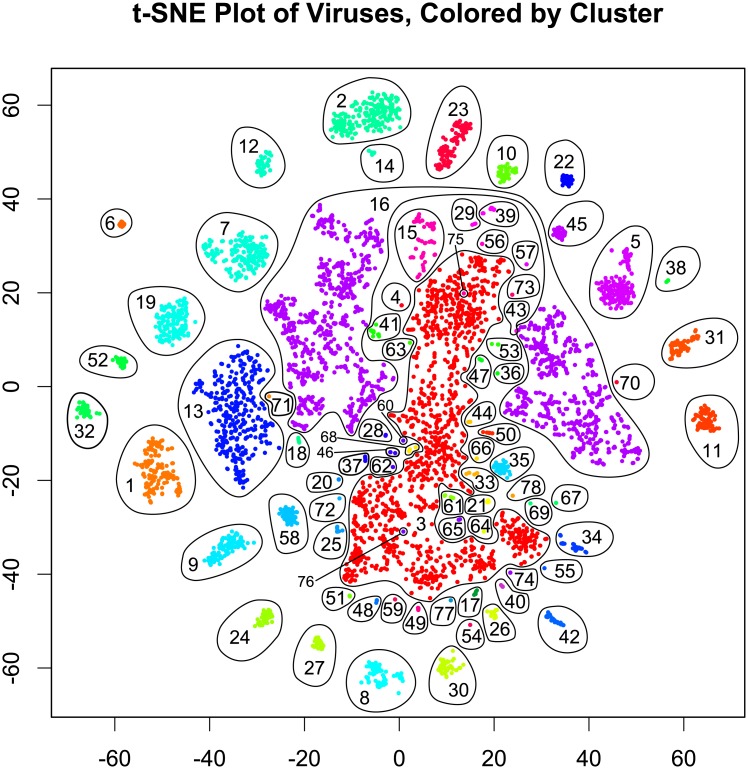
t-SNE plot of 5,817 viruses from RefSeq, grouped by mutual information of genes in common and 4-mer frequency. Points are colored according to 78 clusters assigned by density-based clustering in the 3-dimensional t-SNE space. Each cluster is numbered, and cluster numbers correspond to those in Fig 5. Most clusters correspond to one order or family in the ICTV classification.

In order to further reduce noise while preserving as much structure as possible, we used nonlinear dimensionality reduction. Because our alignment-based method produces more significant results for viruses which are quite similar, we chose to use t-SNE dimensionality reduction, which preserves local structures more than global ones. The t-SNE algorithm is further useful because it relies on the probability of each virus being another’s nearest neighbor, so it is able to discriminate the nearest neighbors equally well for viruses with more genes, which are likely to have more matches. Typically, t-SNE begins by performing principal component analysis to reduce a high-dimensionality system down to 50 dimensions; because we are starting with a distance matrix, rather than a coordinate matrix, we use classical multidimensional scaling to embed the genome space in 50-dimensional Euclidean space (Fig 1(d)) [25]. A Barnes-Hut implementation of t-SNE was used, with perplexity 30 and *θ* = 0.5. The algorithm was run 20 times, and the result with the lowest Kullback-Leibler divergence was chosen. All data analysis was performed on a three-dimensional embedding, whose distances are summarized in Fig 1(e), because the data showed evidence of nontrivial topologies in two dimensions. (For example, note that in Fig 2, cluster 16 is disconnected; although it is shown to be a single cluster by the clustering in three dimensions, it resembles a torus, so a two-dimensional image of it is inadequate.) The utility of t-SNE cannot be extrapolated to more than three dimensions, so a three-dimensional embedding is presumed adequate [26]. This produced clear clusters of various shapes and sizes. Because t-SNE attempts to preserve neighbor probabilities, it suggests a density-based clustering algorithm; for the analysis, we used OPTICS, a variant of DBSCAN which produces hierarchical clustering on the three-dimensional space (Fig 1(f)) [27,28]. This produced results almost identical to those of nearest-neighbor clustering.

## Results and discussion

**Fig 3:**
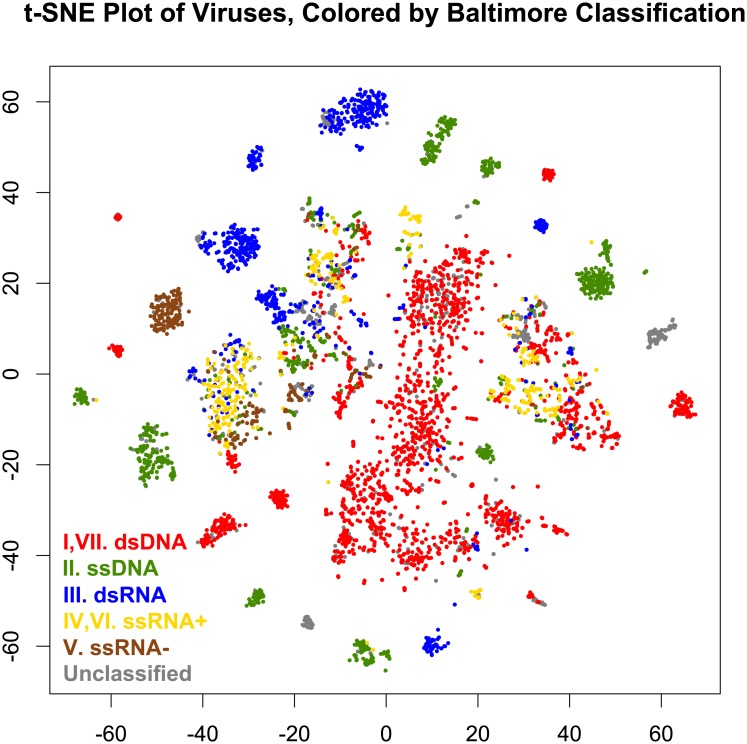
t-SNE plot of 5,817 viruses from RefSeq, grouped by mutual information of genes in common and 4-mer frequency. Points are colored by Baltimore classification: red = dsDNA viruses (Baltimore class I & VII), green = ssDNA viruses (Baltimore class II), blue = dsRNA viruses (Baltimore class III), yellow = ssRNA viruses, positive sense (Baltimore class IV & VI), brown = ssRNA viruses, negative sense (Baltimore class V), and gray = unclassified.

**Fig 4:**
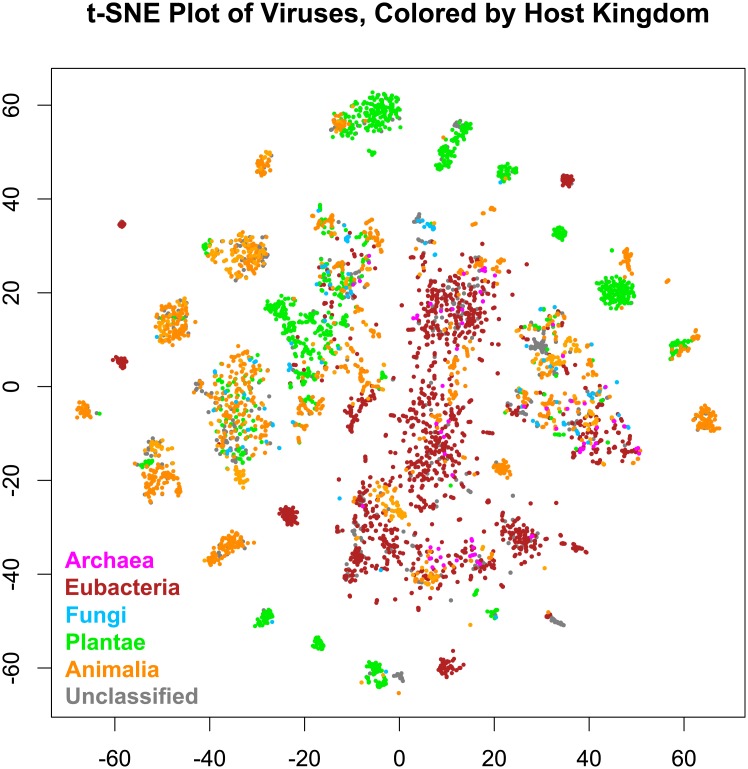
t-SNE plot of 5,817 viruses from RefSeq, grouped by mutual information of genes in common and 4-mer frequency. Points are colored by host kingdom: magenta = Archaea, maroon = Eubacteria, sky blue = Fungi, lime green = Plantae, orange = Animalia, and gray = unclassified.

Fig 2 gives a 2-dimensional t-SNE visualization of the 50-dimensional data, using initial values from a 2-dimensional PCA projection of the 3-dimensional space used for clustering. These are colored by the 78 clusters found in the 3-dimensional t-SNE space. The number of clusters was relatively stable at 78, i.e. a relatively wide range of tree cuts all produced 78 clusters. This is comparable to the 125 families determined by ICTV. Most clusters consist of a single Baltimore type, as shown in Fig 3. Similar results arise when the data are colored by host kingdom instead, as shown in Fig 4.

**Fig 5:**
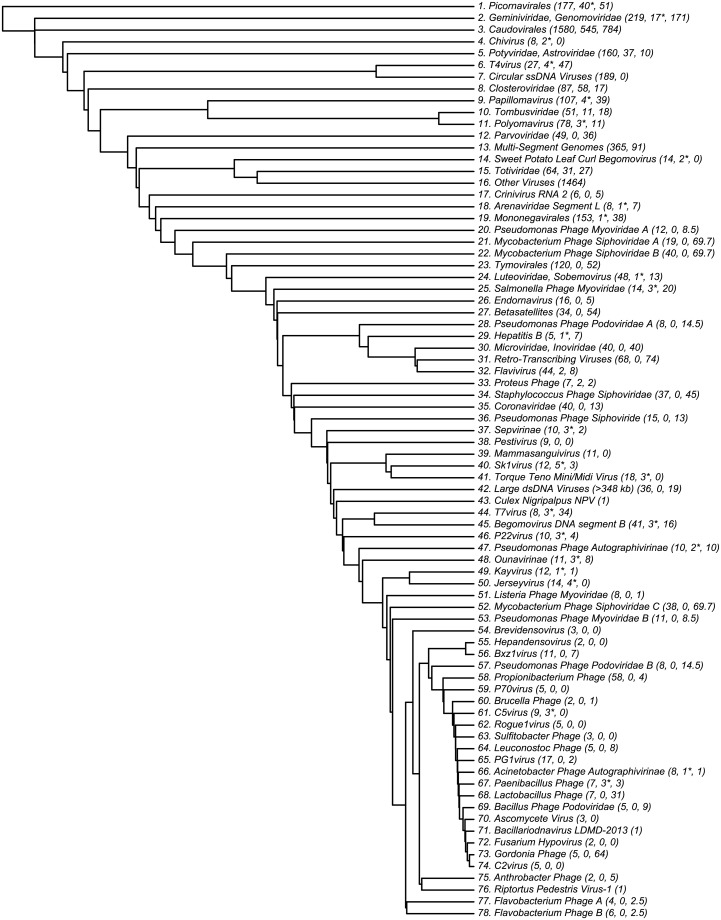
Dendrogram of 78 clusters of viruses, determined by density-based clustering on the 3-dimensional t-SNE space of viral genomes. Cluster numbers correspond to numbers in Fig 4. Three numbers are given in parentheses: the number of viruses in each cluster, the number of viruses which are not part of the family but nevertheless are clustered together with it, and the number of viruses in a family which were not included in its cluster. Asterisks after the second number indicate that most of the incorrect viruses are unclassified by ICTV or otherwise correspond weakly with it. In a few cases, one family was split into multiple clusters; in these cases, the clusters were designated “A,” “B,” etc. If any viruses in the family were missing from all such clusters, their count was divided equally between them, leading to the occasional fractional virus count in the third coordinate. Because this dendrogram was developed from hierarchical clustering, branch lengths correspond to how much more similar a given cluster is to itself than to the other viruses.

In Fig 5, these clusters are shown as a dendrogram. Because hierarchical clustering was used, the branch lengths on this dendrogram indicate how readily a given cluster peels off from the whole. Clusters with longer branches are substantially more similar to themselves than to other clusters according to our algorithm. For example, Polyomavirus (cluster 11) follows Tombusviridae (cluster 10), despite their different Baltimore classes. Branch length is best interpreted as a confidence score in the importance of the cluster in question, rather than a specific evolutionary time measurement.

Almost all clusters could be named using an established ICTV order, family, or genus. Interestingly, cluster 39 was not: it contains a collection of viruses which infect through mammalian blood, such as the Abelson murine leukemia virus, the Hepatitis C viruses, and Pegiviruses. We have named this cluster “Mammasanguiviridae.”

While the Hepatitis C viruses were found in cluster 39, the Hepatitis B viruses displayed significantly broader genetic diversity. Cluster 29 contained all four Hepatitis B viruses which infect bats or monkeys, as well as the bluegill hepadnavirus, an unclassified hepadnavirus which might be considered a Hepatitis B variant with fish as hosts. The strains of Hepatitis B with other hosts were found in various other clusters, predominantly in subcluster 31 of cluster 16. This suggests that for some families of viruses, the specific host may play an important role in determining the virus’s genetics.

While most clusters were mostly or completely unified by a single ICTV class or other feature, cluster 16 (Other Viruses) was not. In three dimensions, cluster 16 forms a ring around cluster 3, suggesting that it may carry a more complicated structure that is not well-represented in the 2-dimensional plot. In order to determine the organization of cluster 16, it is necessary to explore further out on the derived phylogenetic tree. This is done in S3 Fig, which displays a phylogenetic tree of 98 sub-clusters of cluster 16. Distances between viruses in cluster 16 are determined almost completely by the 4-mer variation of information, which yields less conclusive relationships. Most sub-clusters in cluster 16 consist primarily or wholly of one ICTV class, but few contain every element in that class. A few clusters displayed no clear unifying characteristics. The algorithm also tended to elide viroids, satellites, and individual segments of multi-segment genomes; the clusters which lacked unity had large populations of such viruses.

Because of their unique biochemistry, the genetic unity of the retroviruses is also worthy of note. Cluster 31 is made up wholly of retroviruses, suggesting that there are indeed some invariant patterns of expression associated with their transcription process. However, cluster 31 contains only half of the retroviruses in the study; the others are found in several different clusters, such as subcluster 35 of cluster 16, which contains four Polyomaviridae and three retroviruses, all with animals as natural hosts. Unlike most classifications, which rely upon one feature, our taxonomy incorporates predictors of size, biochemistry, host, and other factors, so the final classification can only be explained by a combination of these.

**Fig 6:**
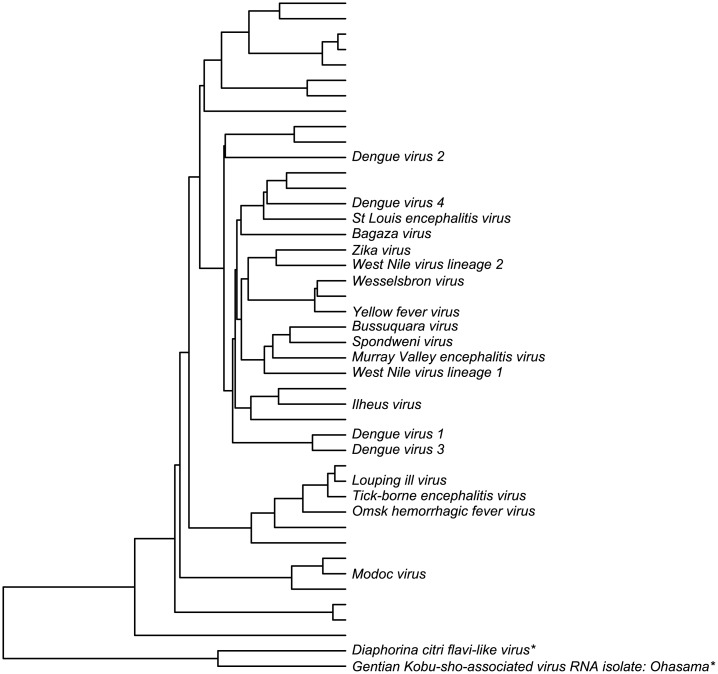
Dendrogram of cluster 32, which corresponds to the Flaviviruses. Of the 44 viruses in this cluster, 19 which are clinically significant in humans are labeled; taxa names for the other 25 are omitted for clarity. This constructed phylogeny suggests that Zika is more genetically similar to the West Nile virus and yellow fever than to the Dengue viruses; the closest Dengue virus is Dengue 4. The two species marked with asterisks are not Flaviviruses; although they were clustered together with them, they are separated from the true Flaviviruses. Eight Flaviviruses make up a single sub-cluster of cluster 16 instead.

Many families of viruses were assigned to clusters consistent with ICTV nomenclature. Of the viruses not classed with cluster 16, 80% were consistently clustered; most of the disagreements (75%) occurred in clusters 3 and 14. As an example, 42 out of 50 of the flavivirus family were found in cluster 12. Of particular interest is the Zika virus and its relation to the Dengue viruses. Traditionally, Zika has been grouped with Spondweni, and believed to be less similar to the four strains of Dengue than they are to each other [29]. However, recent discussion has questioned whether Zika may be more related to some of the Dengue viruses, and whether the differences between the Dengue strains may be more significant [30]. Our analysis is consistent with the traditional approach, as shown in Fig 6. We find Zika to be most genetically similar to the West Nile virus, yellow fever, and Spondweni, than to any of the Dengue viruses. Nevertheless, the Dengue strains are more or less as different from each other as they are from Zika.

This finding merits some additional discussion because our phylogeny does not cluster the four strains of Dengue together, as whole-genome alignment has [29]. In the initial distance matrix, before dimensionality reduction, the Dengue strains are closer to each other than to Zika. Like whole-genome alignment, this distance matrix is more appropriate than our final embedding for comparing closely related sequences. However, it does not produce usable results for building a large-scale taxonomy; in fact, because it does not satisfy the triangle inequality, it does not even permit a concept of proximity. In order to provide a way to compare Dengue with bacteriophage, we are forced to sacrifice some accuracy in comparing Dengue with Zika for greater internal consistency. This is the purpose of the multidimensional scaling and dimensionality reduction: they shave off some of the precision in order to fit all of the viruses into the same global picture. Carrying these steps out is analogous to tuning the focus on a lens: we could focus on very close viruses by using our initial distance matrix, or better yet, whole-genome alignment. Conversely, we could have focused even further out, at the global scale of the Baltimore classification. By using BLAST, k-mers, and statistical processing steps, we focused at an intermediate level between these two extremes, where there has so far been limited quantitative analysis. Ultimately, the available information is limited by the length of the genomes and the heuristic nature of the computations, and the choice of scale is merely a choice of where to concentrate that information, and conversely where to accept imprecision.

By developing a classification which groups viruses into clusters that approximate the size of families, we have developed an impartial comparison which is useful at intermediate scales. At the broadest scale, our classification corroborates the value of the Baltimore classification in dividing viruses objectively into seven discrete groups. When it is possible, whole-genome alignment is still the ideal tool for comparing very similar viruses. For developing intra-family phylogenies, an affinity propagation clustering algorithm (comparable to ours in its quantitative nature, number of simplifications, and relatively rigid clusters) has recently been used to characterize the diversity of rabies viruses [31]. This method assumes more homogenous clusters than ours by relying on a representative sequence for each cluster; ours allows for clusters wherein no virus shares a sequence with every other virus. Our flexibility is a drawback in clustering highly similar viruses (e.g. those of the same family) but advantageous when looking for clusters which trace back to a single ancestor but may evolve divergently. (Imagine, for example, an extinct ancestor with six genes, with 20 living descendants bearing every possible choice of three genes from it: no one descendent shares a gene with every other descendent.) Similarly, vConTACT is a new model for predicting the taxonomy of novel phage genomes with double-stranded DNA, which has been partially validated by its agreement with phenomenological categories [32]. Using a discrete cutoff for BLAST score, vConTACT is best-suited for datasets that admit a black-and-white picture of gene homology [32]. Double-stranded DNA phage do, because horizontal gene transfer plays a major role in their evolution [33]. This gives vConTACT finer-scale fidelity than our model within this subgroup; our model classifies almost all of their viruses into cluster 3. Our grouping fills the mesoscale gap between subgroup analysis (micro) and the Baltimore classification (macro) without resorting to subjective analysis.

We do not intend for our phylogeny to be taken as a new ground truth about the diversity of viruses. Rather, we intend it as an invitation not to become too reliant on existing classifications. Starting with the whole-genome alignment of Needleman and Wunsch, genetic methods have been climbing the phylogenetic tree, and are now able to construct taxonomies between increasingly large and divergent sets of organisms [34]. Genetic methods in taxonomy address continuous variations (down to a single base change), complementing the discrete boundary-drawing of more traditional approaches. The taxonomist draws boundaries, not just between families, but also between the types of similarities which unify an order and those which unify a family or a genus. The present work is an exploration of the use of bioinformatics in this second goal. Rather than establishing discrete categories feature-by-feature (e.g. classifying first by Baltimore class, then by host kingdom within that, then by shape, etc.), our method collects clusters of viruses which are similar by some combination of all of these.

As discussed above, the BLAST score is important for setting the global geography of the virus landscape—not just whether two things are similar or different, but how similar or how different. But once the viral landscape has been mapped, viruses can be placed in the existing structure without computing additional gene alignments. Although the focus of this work is unsupervised learning (developing a new phylogeny), not supervised learning (placing viruses into an existing phylogeny), our model is easily extended to the second task as well. We downloaded the 5,798 most recent virus genomes posted to GenBank at the time of this writing and used a simplified version of the algorithm above to place them into the existing clusters [35]. We computed the k-mer variation of information matrix between the 5,798 new viruses and the 5,817 original viruses. Using a k-nearest neighbors algorithm on these distances, we identified the cluster shared by the plurality of each new virus’s 30 closest neighbors [36,37]. We weighted the count of neighbors in a cluster to favor smaller clusters; otherwise, it would be unreasonably hard for any viruses to be assigned to clusters of less than 15 members, which could never reach a majority of 30.

Unlike RefSeq, GenBank does not have a complete or verified set of metadata corresponding to all genomes. As a result, we will limit ourselves to a few observations. The Flaviviruses are well-differentiated and have been under scrutiny in recent years, and indeed, 537 of the new viruses were clustered with the Flaviviruses in cluster 32, and all of these are well-labeled in GenBank. Of these, 356 are titled in GenBank with ICTV Flavivirus names. If we rank these by the percentage of the weighted count of nearest neighbors held by the assigned cluster, we find that 207 viruses which best agree with cluster 32 are all Flaviviruses, and of the 122 which least agree with cluster 32 (but were still assigned to it), none are Flaviviruses. This suggests some consistency within our model: it is most accurate about the viruses for which it has the least ambiguous result. By setting a threshold and allowing some viruses not to be counted as part of a cluster at all, one could build more homogeneous clusters and even determine when the data suggest forming additional clusters, as carried out in GRAViTy [13]. Classifying viruses phenomenologically is faster when an existing tree, with the relevant features of each branch, is already in place; so also, classifying viruses genetically can be carried out quickly once the geography of the diversity of viruses is mapped.

Future work could focus on developing new mathematical models for genetic similarity or faster or more accurate alignment tools. Nevertheless, our classification has theoretical motivation, is validated empirically, and only takes a few days to run the entire analysis on standard computing resources. Incremental viruses could be added without having to re-compute the whole structure because they would not change it measurably. However, the greatest contributions to viral phylogeny will be the sequencing of many new viruses, in order to better understand the structure of the phylogenetic tree as a whole.

In conclusion, we have shown that a genetic classification using only quantitative features can provide a meaningful viral taxonomy. This represents a significant advancement over previous alignment-free schemes which lack theoretical motivation and which, despite producing similarity metrics, have yet to be carried out to the extent needed to attempt a global phylogeny. It offers advantages over qualitative classification by virologists because it is not subjective. Our algorithm provides an example of a mathematical model which could be used to offer an impartial check on classifications constructed by humans, and form a starting point for quantitative models in other areas of genetics and evolution. Because of its theory and validation, our model presents a compelling case for stronger reliance on quantitative genomics across the studies of virology and taxonomy.

## Supporting information

S1 Fig Plot of data classified using only the gene alignment distance. The cluster labels and colors are the same as in Fig 4. Notice that although many of the same structures are present, there is little distinction between smaller and larger groupings in the middle.
(EPS)

S2 Fig Plot of data classified using only the 4-mer distance. The cluster labels and colors are the same as in Fig 4. Although the 4-mer distance is able to provide some measure of similarity between very similar and very different sequences, it is not well-suited for an overall classification.
(EPS)

S3 Fig Dendrogram of 98 sub-clusters of cluster 16. The number of viruses in each cluster is given in parentheses. Relationships within sub-clusters are weaker, and a few clusters (those for which no name is given) displayed no unifying characteristics.
(EPS)

## References

[1] Moreira D, López-García P. Ten reasons to exclude viruses from the tree of life. Nature Reviews Microbiology. 2009;7: 306–311. 10.1038/nrmicro2108 19270719

[2] Foulongne V, Sauvage V, Hebert C, Dereure O, Cheval J, Gouilh MA, et al Human Skin Microbiota: High Diversity of DNA Viruses Identified on the Human Skin by High Throughput Sequencing. PLOS ONE. 2012;7: e38499 10.1371/journal.pone.0038499 22723863 PMC3378559

[3] Pietilä MK, Roine E, Paulin L, Kalkkinen N, Bamford DH. An ssDNA virus infecting archaea: a new lineage of viruses with a membrane envelope. Molecular Microbiology. 2009;72: 307–319. 10.1111/j.1365-2958.2009.06642.x 19298373

[4] Lefkowitz EJ, Dempsey DM, Hendrickson RC, Orton RJ, Siddell SG, Smith DB. Virus taxonomy: the database of the International Committee on Taxonomy of Viruses (ICTV). Nucleic Acids Res. 2018;46: D708–D717. 10.1093/nar/gkx932 29040670 PMC5753373

[5] Siddell SG, Walker PJ, Lefkowitz EJ, Mushegian AR, Adams MJ, Dutilh BE, et al Additional changes to taxonomy ratified in a special vote by the International Committee on Taxonomy of Viruses (October 2018). Arch Virol. 2019;164: 943–946. 10.1007/s00705-018-04136-2 30663020

[6] Mahmoudabadi G, Phillips R. A comprehensive and quantitative exploration of thousands of viral genomes. eLife. 2018;7: e31955 10.7554/eLife.31955 29624169 PMC5908442

[7] Pedulla ML, Ford ME, Houtz JM, Karthikeyan T, Wadsworth C, Lewis JA, et al Origins of Highly Mosaic Mycobacteriophage Genomes. Cell. 2003;113: 171–182. 10.1016/s0092-8674(03)00233-2 12705866

[8] Simmonds P, Adams MJ, Benkő M, Breitbart M, Brister JR, Carstens EB, et al Consensus statement: Virus taxonomy in the age of metagenomics. Nature Reviews Microbiology. 2017;15: 161–168. 10.1038/nrmicro.2016.177 28134265

[9] Pruitt KD, Tatusova T, Maglott DR. NCBI reference sequences (RefSeq): a curated non-redundant sequence database of genomes, transcripts and proteins. Nucleic Acids Research. 2006;35: D61–D65. 10.1093/nar/gkl842 17130148 PMC1716718

[10] RefSeq: NCBI Reference Sequence Database [Internet]. [cited 20 May 2019]. https://www.ncbi.nlm.nih.gov/refseq/

[11] Yu C, Hernandez T, Zheng H, Yau S-C, Huang H-H, He RL, et al Real Time Classification of Viruses in 12 Dimensions. PLOS ONE. 2013;8: e64328 10.1371/journal.pone.0064328 23717598 PMC3661469

[12] Hoang T, Yin C, Zheng H, Yu C, Lucy He R, Yau SS-T. A new method to cluster DNA sequences using Fourier power spectrum. Journal of Theoretical Biology. 2015;372: 135–145. 10.1016/j.jtbi.2015.02.026 25747773 PMC7094126

[13] Aiewsakun P, Simmonds P. The genomic underpinnings of eukaryotic virus taxonomy: creating a sequence-based framework for family-level virus classification. Microbiome. 2018;6: 38 10.1186/s40168-018-0422-7 29458427 PMC5819261

[14] Rohwer F, Edwards R. The Phage Proteomic Tree: a Genome-Based Taxonomy for Phage. Journal of Bacteriology. 2002;184: 4529–4535. 10.1128/JB.184.16.4529-4535.2002 12142423 PMC135240

[15] Cover TM, Thomas JA. Elements of Information Theory. John Wiley & Sons; 2012.

[16] Altschul SF, Gish W, Miller W, Myers EW, Lipman DJ. Basic local alignment search tool. J Mol Biol. 1990;215: 403–410. 10.1016/S0022-2836(05)80360-2 2231712

[17] Altschul SF, Madden TL, Schäffer AA, Zhang J, Zhang Z, Miller W, et al Gapped BLAST and PSI-BLAST: a new generation of protein database search programs. Nucleic Acids Res. 1997;25: 3389–3402. 10.1093/nar/25.17.3389 9254694 PMC146917

[18] Gish W, States DJ. Identification of protein coding regions by database similarity search. Nat Genet. 1993;3: 266 10.1038/ng0393-266 8485583

[19] Camacho C, Coulouris G, Avagyan V, Ma N, Papadopoulos J, Bealer K, et al BLAST+: architecture and applications. BMC Bioinformatics. 2009;10: 421 10.1186/1471-2105-10-421 20003500 PMC2803857

[20] Mazandu GK, Mulder NJ. Scoring Protein Relationships in Functional Interaction Networks Predicted from Sequence Data. PLOS ONE. 2011;6: e18607 10.1371/journal.pone.0018607 21526183 PMC3079720

[21] Korf I, Yandell M, Bedell J. BLAST. O’Reilly Media, Inc.; 2003.

[22] Ounit R, Wanamaker S, Close TJ, Lonardi S. CLARK: fast and accurate classification of metagenomic and genomic sequences using discriminative k-mers. BMC Genomics. 2015;16: 236 10.1186/s12864-015-1419-2 25879410 PMC4428112

[23] Dubinkina VB, Ischenko DS, Ulyantsev VI, Tyakht AV, Alexeev DG. Assessment of k-mer spectrum applicability for metagenomic dissimilarity analysis. BMC Bioinformatics. 2016;17: 38 10.1186/s12859-015-0875-7 26774270 PMC4715287

[24] Anwar F, Baker SM, Jabid T, Mehedi Hasan M, Shoyaib M, Khan H, et al Pol II promoter prediction using characteristic 4-mer motifs: a machine learning approach. BMC Bioinformatics. 2008;9: 414 10.1186/1471-2105-9-414 18834544 PMC2575220

[25] Leeuw J de, Mair P. Multidimensional Scaling Using Majorization: SMACOF in R. 2011; https://escholarship.org/uc/item/9z64v481

[26] van der Maaten L, Hinton G. Visualizing Data using t-SNE. Journal of Machine Learning Research. 2008;9: 2579–2605.

[27] Ankerst M, Breunig MM, Kriegel H, Sander J. OPTICS: Ordering points to identify the clustering structure. ACM Press; 1999. pp. 49–60.

[28] Ester M, Kriegel H-P, Sander J, Xu X. A density-based algorithm for discovering clusters in large spatial databases with noise. AAAI Press; 1996. pp. 226–231.

[29] Kuno G, Chang G-JJ, Tsuchiya KR, Karabatsos N, Cropp CB. Phylogeny of the Genus Flavivirus. Journal of Virology. 1998;72: 73–83. 9420202 10.1128/jvi.72.1.73-83.1998 PMC109351

[30] Kelser EA. Meet dengue’s cousin, Zika. Microbes and Infection. 2016;18: 163–166. 10.1016/j.micinf.2015.12.003 26706817

[31] Fischer S, Freuling CM, Müller T, Pfaff F, Bodenhofer U, Höper D, et al Defining objective clusters for rabies virus sequences using affinity propagation clustering. PLOS Neglected Tropical Diseases. 2018;12: e0006182 10.1371/journal.pntd.0006182 29357361 PMC5794188

[32] Bolduc B, Jang HB, Doulcier G, You Z-Q, Roux S, Sullivan MB. vConTACT: an iVirus tool to classify double-stranded DNA viruses that infect Archaea and Bacteria. PeerJ. 2017;5: e3243 10.7717/peerj.3243 28480138 PMC5419219

[33] Hendrix RW, Smith MCM, Burns RN, Ford ME, Hatfull GF. Evolutionary relationships among diverse bacteriophages and prophages: All the world’s a phage. PNAS. 1999;96: 2192–2197. 10.1073/pnas.96.5.2192 10051617 PMC26759

[34] Needleman SB, Wunsch CD. A general method applicable to the search for similarities in the amino acid sequence of two proteins. Journal of Molecular Biology. 1970;48: 443–453. 10.1016/0022-2836(70)90057-4 5420325

[35] Clark K, Karsch-Mizrachi I, Lipman DJ, Ostell J, Sayers EW. GenBank. Nucleic Acids Res. 2016;44: D67–D72. 10.1093/nar/gkv1276 26590407 PMC4702903

[36] Fix E, Hodges J. Discriminatory Analysis—Nonparametric Discrimination: Consistency Properties [Internet]. CALIFORNIA UNIV BERKELEY; 1951 Feb. https://apps.dtic.mil/docs/citations/ADA800276

[37] Cover T, Hart P. Nearest neighbor pattern classification. IEEE Transactions on Information Theory. 1967;13: 21–27. 10.1109/TIT.1967.1053964

